# Ultra-low-dose radiotherapy in the treatment of ocular adnexal lymphoma: a prospective study

**DOI:** 10.1186/s13014-022-02180-6

**Published:** 2022-12-20

**Authors:** Xiaolu Yang, Ruonan Wang, Xiaochun Yuan, Shengyu Yao, Chungang Wang, Jinwei Cheng

**Affiliations:** 1 Department of Ophthalmology, Shanghai General Hospital, Shanghai JiaoTong University, Shanghai, China; 2 Shanghai Key Laboratory of Ocular Fundus Disease, Shanghai, China; 3 Shanghai Engineering Center for Visual Science and Photomedicine, Shanghai, China; 4 National Clinical Research Center for Eye Diseases, Shanghai, China; 5 Shanghai Engineering Center of Precise Diagnosis and Treatment of Eye Diseases, Shanghai, China; 6 Department of Radiology, Shanghai General Hospital, Shanghai Jiaotong University, Shanghai, China; 7 Department of Radiation Oncology, Shanghai General Hospital, Shanghai Jiaotong University, No.100, Haining Road, Shanghai, 200080 China; 8 Department of Ophthalmology, Eye and ENT Hospital, Fudan University, Shanghai, China

## Abstract

**Purpose:**

This single-arm, prospective, exploratory study investigated the effectiveness of ultra-low-dose radiotherapy in the treatment of ocular adnexal lymphoma (OAL).

**Patients and methods:**

Patients with pathologically confirmed ocular adnexal low-grade non-Hodgkin lymphoma (predominantly mucosa-associated lymphoid tissue, MALT or follicular lymphoma) were included and treated with ultra-low-dose radiotherapy consisting of 2 successive fractions of 2 Gy at our institution between 2019 and 2021. Disease response was assessed clinically and radiographically within 4 months and at 3 to 6-month intervals after treatment. Data collected included rates of overall response, complete response (CR), partial response (PR), lesion size, and acute/chronic ocular toxic effects.

**Results:**

Sixteen patients with median age of 63 years (range 23–86 years) were included in the study. The histological subtypes included MALT (11 patients; 69%); follicular lymphoma (2 patients; 12%); Lymphoid hyperplasia (3 patient, 19%). At a median follow-up time of 15.5 months (range 5.0–30.0 months), the overall response rate was 88%, with a CR rate of 75% (n = 12) and a PR rate of 13% (n = 2). The average lesion area was reduced from 117.9 ± 60.4 mm^2^ before radiation therapy to 38.7 ± 46.0mm^2^ at initial evaluation post radiation therapy (*P* = 0.002, n = 16), and to 8.5 ± 21.2 mm^2^ (*P* < 0.001 compared with postoperative lesion area) in patients with response at one year (n = 11). Disease progression was noted in 2 patients (12%). The 1-year rates of local progression-free survivals (LPFS) and overall survival (OS) were 85% and 100%, respectively. No distant relapses were observed in any of the patients. No acute or late toxic effects were noted.

**Conclusion:**

Ultra–low-dose radiotherapy in patients with OAL is associated with excellent local disease control and long-term survival with no significant acute or late toxicities.

**Supplementary Information:**

The online version contains supplementary material available at 10.1186/s13014-022-02180-6.

## Introduction

Ocular adnexal lymphoma (OAL) includes lesions of the conjunctiva, lacrimal gland, orbital soft tissue, or eyelid [1]. Compared with other sites in the body, ocular adnexal involvement is relatively rare, accounting for 5–10% of all extranodal lymphomas [2]. But it represents the most common primary orbital malignancy in adults [3–5]. And the incidence of OAL has been noted to be significantly increasing, with annual rates ≥ 6% [6, 7].

This malignancy is predominantly low-grade non-Hodgkin lymphoma (NHL). The most common histologic subtypes of OAL are extranodal marginal-zone B cell lymphoma (EMZL) originating in the mucosa-associated lymphoid tissue (MALT), followed by follicular lymphoma (FL), mantle cell lymphoma (MCL) and diffuse large B cell lymphoma (DLBCL) [1].

Treatment modalities for OAL include observation, radiotherapy, surgery, and systemic chemotherapy. While standard-of-care management of stage I and II low-grade OAL remains external-beam radiotherapy (EBRT), with studies generally demonstrating local control rates over 90–100% after conventional radiation with doses of 24–40 Gy [8–11]. Radiation-related side effects were tolerable but with increasing severe complications (grade 3–4) when more than 36 Gy is administered, including keratitis, severe dry eye syndrome, glaucoma, retinopathy, and cataract formation [11–13].

More recent studies have assessed the efficacy of lower doses of radiotherapy, in an attempt to minimise radiotherapy related side effects to the orbit without compromising local control [14, 15]. Fasola et al. first reported, in 2013, in a study of 27 sites of ocular adnexal involvement in 20 patients with non-Hodgkin lymphoma treated with ultra-low-dose radiotherapy (RT), defined as a total radiation dose of 4 Gy delivered in two successive fractions of 2 Gy each [14]. Complete response was achieved in 23 sites (85%), partial response in 3 sites (11%), and only 1 site (4%) required full-dose therapy. We reported a case series of biopsy-proven choroidal lymphoma treated with ultra-low-dose radiotherapy with complete tumor regression and no toxic effects [16].

Against these retrospective studies confirming that 4 Gy in two fractions was an effective dose in the local treatment of OAL,we did a prospective study in patients with OAL at our institution, evaluating the efficacy and toxicity of ultra-low-dose RT in the treatment of OAL based on detailed clinical examination and radiographic imaging measurements, with the aim of showing that a lower dose of 4 Gy in two fractions is also safe and effective in terms of local control of OAL.

## Material and methods

### Participants and study design

This is a single-arm, prospective, exploratory study evaluating the efficacy of ultra-low-dose RT in the treatment of OAL.

Patients were eligible for this study on the basis of the following criteria: (1) patients with pathologically confirmed ocular adnexal low-grade NHL (MALT or follicular lymphoma); (2) patients who consented to received a ultra-low-dose RT course (4 Gy in two fractions over 2 days); (3) patients who received no other therapy concomitantly with ultra-low-dose RT until evaluation of the response.

Full workup consisted of a comprehensive ocular examination by two licensed ophthalmologists (X.Y. and J. C.), basic laboratory studies, and baseline imaging (positron emission tomography-CT or ultrasound imaging of the neck, abdomen, and pelvis). The orbits were imaged with either CT (1 patient with internal metallic plate) or MRI (15 patients). Preoperative and postoperative MRI was acquired by use of a 1.5-T MRI unit (Discovery MR750W; GE Medical Systems). Coronal, axial and sagittal plane T1-weighted and fat-suppressed T2-weighted images of the orbit were obtained. All sequences used a 256 × 256 matrix, a 28 cm field of view and slice thickness of 1.0 mm.

All patients were staged clinically according to the Ann Arbor staging system. Patients with synchronous bilateral ocular adnexal involvement in the absence of distant lymphoma were considered to have stage IE disease.

Shanghai General Hospital Research Ethics committee reviewed the study and all patients provided written informed consent (authorization code [2019]72 and approval date Oct.20, 2019). The work was done under strict Health Insurance Portability and Accountability Act compliance.

### Treatment

All patients were immobilized with a custom thermoplastic mask and received computed tomography (CT) scans on a Brilliance BigBore scanner (Philips Healthcare, Andover, MA, USA) with a 1 mm slice thickness. The CT images were transferred to treatment planning system (Eclipse Version 13.6, Varian Medical Systems Inc., Palo Alto, CA) for volumetric modulated arc therapy (VMAT) plan generation. The clinical target volume (CTV) was defined as the specific orbit lesion. The CTV-to-planned target volume (PTV) treatment margins were 2 mm. The prescription dose was delivered to CTV by 4 Gy in 2 fractions for first line treatment, and additional 24 Gy in 12 fractions for cases with local recurrence. Plans were generated for an EDGE linear accelerator (Varian Medical Systems, Palo Alto, CA) using 6 MV photons with high-definition multi-leaf collimator(MLC). 2 partial arcs rotating from 260° to 100° clockwise then back were used. Five patients with bilateral lesions were treated simultaneously.

All patients underwent excisional biopsies prior to RT. Radiothearpy was given when ocular adnexal low-grade NHL was pathologically confirmed. Time duration between operation and RT was usually within one month.

### Response assessment

Initial response was assessed with clinical examination and radiographic imaging within 4 months with the same imaging modality used at diagnosis to define the lymphoma. Given the low yield of MRI for the identification of conjunctival lymphoma, baseline and response assessments were clinical for patients with conjunctival lesion who did not have a detectable radiographic abnormality on baseline evaluation. PET-CT was not required for response assessment. But we suggested our patients to have systemic workup annually and continued surveillance by an oncologist to rule out any systemic diseases.

One independent radiologist (X.Y.) who was not involved in patient care and was blinded to the outcome of the lesion evaluation assessed the MRI. We carefully examed every slice in the coronal, transverse or sagittal planes, identified and calculated the lesion area on the slice with maximum dimension. The margins of the lesion were marked manually and calculated using GE ADW4.3 Workstation (GE Medical Systems).

### Toxicity

Acute and chronic ocular toxic effects were assessed and graded according to the Radiation Therapy Oncology Group (RTOG) acute/late radiation morbidity scoring criteria [17]. We monitored patients for acute ocular toxic effects before and during radiotherapy, and at 4 weeks after radiotherapy. We assessed chronic ocular toxic effects at 3 months and 6 months after radiotherapy, and thereafter at 6-month intervals.

### Statistical analysis

Complete response (CR) was defined as resolution of tumor by clinical examination and by radiographic studies. Partial response (PR) was defined as a decrease in size of the disease burden with radiographic studies. Progressive disease was defined as any increase in OAL on clinical examination or imaging studies. Patients who did not progress within the irradiated field were censored at death or the date last seen. The overall response rate was defined as the rate of CR and PR. In order to avoid inter-eye correlation, only one eye was included in the study. For patients with bilateral lesions, the eye with bigger lesion was set as the study eye.

Overall survival (OS) was calculated in months from the beginning of radiotherapy until the last date of follow-up or death. Local progression-free survival (LPFS) was calculated in months from the beginning of radiotherapy until the diagnosis of recurrent disease of the orbit or elsewhere.

The survival rates were displayed using the Kaplan–Meier method. All statistical analyses were performed using the software SPSS 20.0 (IBM Corporation, Armonk, NY USA).

## Results

A total of 16 patients were identified and included in this study. Patient characteristics are detailed in Table 1. The median age for OAL was 63 years (range 23–86 years). There were 9 males (56%) and 7 females (44%). Of all 16 patients for which tumor biopsies were done and diagnoses were given, 11 (69%) were MALT, 2 (12%) were follicular lymphoma and 3 (19%) were reactive lymphoid hyperplasia (Table 1). Since there has been a shift in diagnostic patterns with previously thought benign reactive lymphoid hyperplasia (RLH) being reclassified as extranodal marginal zone lymphoma (ENMZL) [18], three patients with RLH was also included in the study. Five patients (31%) presented with bilateral orbital involvement, and six patients had multiple sites of involvement in the same eye. The orbit (n = 11, 41%) was the most frequent site of OAL, with 5 lymphomas involving the conjunctiva (n = 5, 19%), and the eyelid (n = 5, 19%), followed by rectus (n = 4, 14%), and the lacrimal gland (n = 2, 7%) (some patients had involvement of more than one anatomical structure). All of the five cases of bilateral involvement were treated simultaneously. All patients were of a good performance status (Karnofsky Performance Status Scale 90–100).

**Table 1:** Clinical characteristics of OAL patients

Characteristics	Patients (n = 16), n (%)
Age at presentation (y)	
Median (range)	63 (23–86)
≤ 60 years	6 (37)
> 60 years	10 (63)
Sex	
Male	9 (56)
Female	7 (44)
Histologic subtype	
MALT	11 (69)
FL	2 (12)
Lymphoid Hyperplasia	3 (19)
Laterality	
Unilateral	11 (69)
Bilateral	5 (31)
Site of involvement	Sites (n = 27), n (%)
Orbital soft tissue	11 (41)
Conjunctiva	5 (19)
Lacrimal gland	2 (7)
Eyelid	5 (19)
Rectus	4 (14)
Initial symptoms*	
Eyelid swelling	7 (33)
Proptosis	11 (52)
Ptosis	1 (5)
Eye redness	1 (5)
Blurred vision	3 (14)

*MALT* mucosa-associated lymphoid tissue; *FL* follicular lymphoma

^*^Multiple symptoms possible

**Fig. 1:**
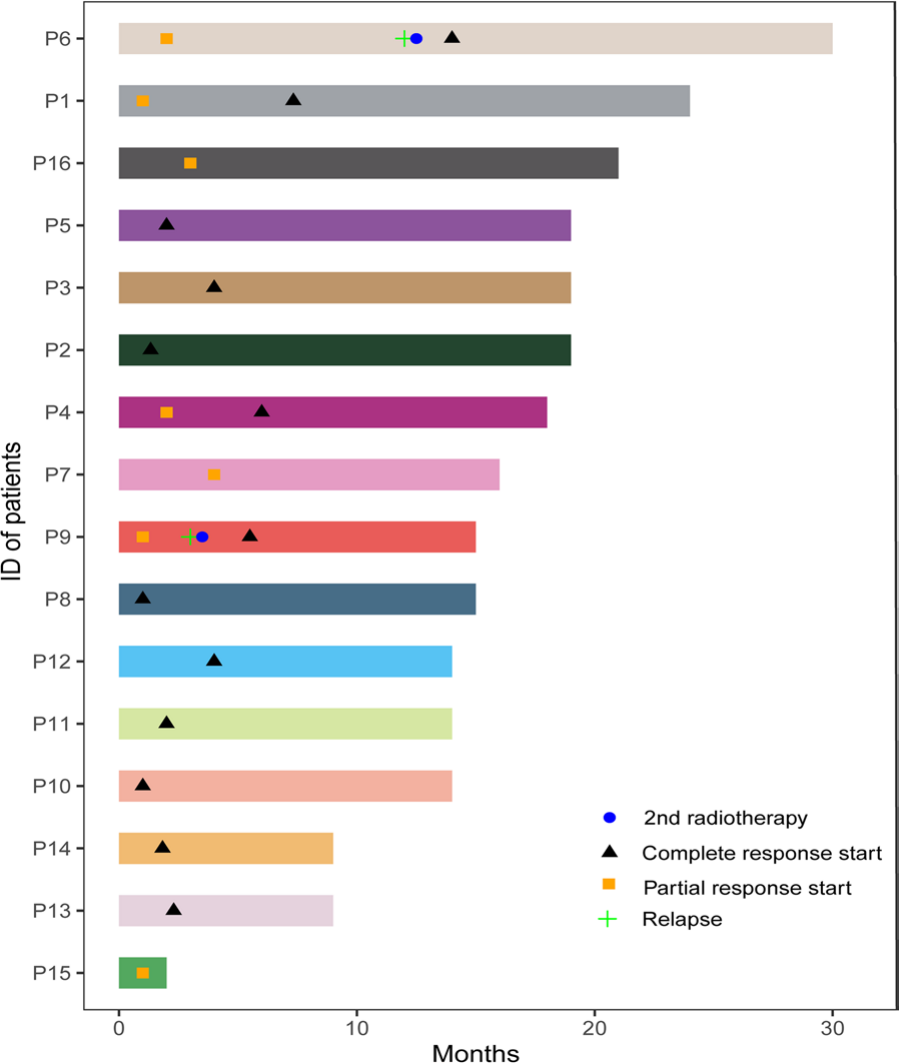
Rate of progression-free survival and overall survival among patients with ocular adnexal lymphoma who was treated with ultra-low-dose radiotherapy (n = 16)

Treatment outcomes are outlined in Table 2. The median follow-up was 15.5 months (range 5.0—30.0 months). Initial response was assessed within 4 months after completion of RT. All sixteen patients had an initial response at a median of 2.0 months (range 1.0–4.0 months). CR was seen in 9 of the 16 patients (56%) and RP was seen in 7 patients (44%). Of the 7 patients with PR at initial evaluation, 3 patients eventually achieved a CR at a median of 6.0 months (range, 5.0—7.3 months) after RT and 2 patients had recurrence at a median of 7.5 months (range, 3.0–12.0 months). Representative examples of treatment outcomes in two patients are shown in Additional file 1: Figs. S1 and S2. Ultimately, the overall response rate (CR and PR) for 16 patients was 88%. CR was observed in 12 (75%) patients and PR was observed in 2 (13%) patients. Of the 12 patients with CR, the median time to CR from RT start date was 2 months (range 1–7.3 months) and median duration 14 months (range 0—19 months). Time to response for each patient was depicted in Fig. 1.

**Table 2:** Clinical outcomes of patients with OAL treated with ultra-low-dose RT

Comm treatment	Patients (n = 16), n (%)
Follow-up Time (mo)	
Median (range)	15.5 (5.0–30.0)
Best corrective visual acuity	
Baseline, Median (range)	20/35 (20/20-LP)
Final, Median (range)	20/30 (20/20-HM)
Lesion Area (mm^2^)	
Baseline	203.8 ± 92.2
Post-operation	117.9 ± 60.4*
Initial evaluation post-RT	38.7 ± 46.0^#^
One year post RT (n = 13)	
With regression (n = 11)	8.5 ± 21.2^#^
With recurrence (n = 2)	191.7 ± 13.9
Local response at initial evaluation (n = 16)	
Complete regression	9 (56)
Partial regression	7 (44)
Progression	0 (0)
Local response at 6 months (n = 14)	
Complete regression	11 (79)
Partial regression	2 (14)
Progression	1 (7)
Time to Complete regression (mo)	
Median (range)	2.0 (1.0–7.3)
Time to recurrence (mo)	
Median (range)	7.5 (3.0–12.0)
Disease status at last follow-up	
Complete regression	13 (81)
Alive with disease	3 (19)
Dead due to lymphoma	0 (0)
Dead due to causes other than lymphoma	0 (0)
Progression-free survival at one year (%)	85
Progression-free survival (mo)	
Median (range)	15.5 (5.0–24.0)
Ocular toxicity (n = 16)	
Acute toxic effect	0 (0)
Chronic toxic effect	0 (0)

*OAL* ocular adnexal lymphoma, *RT* radiation therapy

^*^P < 0.01 compared with baseline lesion area; ^#^*P* < 0.01 compared with postoperative lesion area

Disease progression was noted in 2 patients (12%). A patient (case 9) with MALT lymphoma of the orbital soft tissue experienced an initial regression of the lesion from lesion area of 185.8–68.5 mm^2^ at one-month after RT. Local recurrence developed 3 months after completion of ultra–low-dose RT. The other patient (case 6) with MALT lymphoma of the orbital soft tissue lost follow-up after completion of ultra–low-dose RT and developed a relapse 12 months after. Those two patients were retreated with conventional dose radiation (24 Gy in 12 fractions) and CR was achieved within a median follow-up time of 15 months (range 12–18 months). The 1-year rates of LPFS and OS were 85% and 100%, respectively (Table 2). No distant relapses was observed in any of the patients.

We also evaluated treatment response based on radiographic imaging measurements. All patients underwent excisional biopsies prior to RT. The average baseline lesion area was 203.8 ± 92.2 mm^2^, the average postoperative lesion area was 117.9 ± 60.4 mm^2^ (*P* = 0 0.001). The average post RT lesion area was reduced to 38.7 ± 46.0 mm^2^ (*P* = 0.002, compared with postoperative lesion area) at initial evaluation. At one-year follow-up, the average lesion area was 8.5 ± 21.2 mm^2^ (*P* < 0.001 compared with postoperative lesion area) in patients with response (n = 11) and 191.7 ± 13.9 mm^2^ (*P* = 0.115 compared with postoperative lesion area) in patients with recurrence (n = 2).

We monitored patients for acute and chronic ocular toxic effects and graded according to RTOG acute/late radiation morbidity scoring criteria. Ultra–low-dose RT was well tolerated. No acute or late toxic effects were noted (Tables 3, 4).

**Table 3:** Summary of patient case details

Patient No./ Sex/Age(yrs)/Study Eye	Duration ofSymptoms (mos)	Histological Subtype	Site of Involvement	Visual Acuity		Lesion Area (mm^2^)				Local Response	Follow-up (mos)
				Initial	Final	Baseline	Post-operation	Intial Evaluation post RT	One year post RT		
1/F/63/OS	2	MALT	Conjunctiva Rectus	20/50	20/50	237.9	138.5	69.7	0	CR	24
2/M/42/OD*	6	MALT	Lacrimal gland Rectus	20/20	20/20	282.3	57.1	0	0	CR	19
223/M/52/OD	12	MALT	Conjunctiva Eyelid Rectus	20/20	20/20	85.5	85.5	52.4	0	CR	24
4/F/76/OD	1	Lymphoid hyperplasia	Orbit Eyelid	20/60	20/60	177.5	100.6	68.1	0	CR	18
5/F/67/OD	1	MALT	Eyelid	20/40	20/25	288.4	126.3	0	0	CR	19
6/M/66/OS	3	MALT	Orbit	20/20	20/20	280.7	204.7	164.8	181.8	Progression	30
7/F/86/OD	4	FL	Orbit Rectus	LP	HM	352.7	172.3	68.1	68.1	PR	16
8/F/61/OD	2	Lymphoid hyperplasia	Orbit	20/30	20/30	243.5	78.2	0	0	CR	15
9/M/52/OS	2	MALT	Eyelid Orbit	20/20	20/20	185.8	185.8	68.5	201.5	Progression	15
10/M/84/OD	2	MALT	Orbit	20/50	20/50	168.2	53.6	0	0	CR	14
11/M/62/OS*	12	MALT	Orbit	20/30	20/30	63.7	63.7	0	0	CR	14
12/F/45/OD*	2	MALT	Orbit	20/60	20/60	54.3	54.3	27.9	0	CR	14
13/M/69/OS	7	FL	Conjunctiva Orbit	20/40	20/40	139.6	78.2	0	/	CR	9
14/F/23/OD*	3	MALT	Conjunctiva Orbit	20/20	20/20	128.2	128.2	0	/	CR	9
15/M/68/OD	11	MALT	Conjunctiva Eyelid Orbit	20/40	20/40	257.5	257.5	74.4	/	CR	5
16/M/50/OD*	6	Lymphoid hyperplasia	Lacrimal gland	20/20	20/20	315	101.3	25.1	25.1	PR	21

*M* male, *F* female, *MALT* mucosa-associated lymphoid tissue, *FL* follicular lymphoma, *RT* radiation therapy, *CR* complete response, *PR* partial response

^*^For patients with bilateral lesions, the eye with bigger lesion was set as the study eye

**Table 4:** Studies comparing clinical outcomes and radiotherapy-related toxicities in patients with OAL treated with ultra-low-dose radiotherapy

Study, year (ref)	Clinical characteristics	Treatment	Number of study patients	Disease outcome at follow-up		Type(s) of side effects	No. of patients with side effects
				Partial response (PR) rate	complete response (CR) rate		
[14]	Indolent non-Hodgkin lymphoma (NHL) of the ocular adnexa	2 fractions of 2 Gy	20	11%	85%	Dry eye	1
						Acute conjunctivitis	1
						Transient periorbital edema	4
[15]	Indolent orbital lymphomas	2 fractions of 2 Gy	7	0%	100%	Xerophthalmia	1
[20]	B-cell ocular adnexal lymphoma	2 fractions of 2 Gy	22	14%	86%	Dry eye	1
This study	Ocular adnexal low-grade non-Hodgkin lymphoma	2 fractions of 2 Gy	16	13%	75%	none	none

## Discussion

In this report, we conducted a prospective study evaluating the efficacy of ultra–low-dose RT for low-grade ocular adnexal lymphoma in 16 patients. These patients achieved local control rates of 88%.

The standard RT dose for low-grade OAL ranged from 24–40 Gy, with reported local control rates over 90% [8, 9]. However, a range of acute and late side effects associated with the treatment has been reported. These include dry eye, keratitis, cataract formation, and retinal damage. Goda et al. documented the late side effects in 40 out of 89 orbital MALT lymphoma patients who received radiotherapy with a dose of 25–30 Gy [19]. Cataracts were observed in 22 patients (cumulative incidence of grade 3 cataract requiring surgery was 25% at 7 years), dry eye(s) in 22, keratitis in 3, and macular degeneration/cystoid edema in 2 patients.

Given the radiosensitivity of indolent lymphoma balanced against risk of radiation toxicity, lower doses of radiation have been explored for ocular adnexal lymphoma with favorable local tumor control and minimal toxicity [13, 20].

Several retrospective works suggested that 4 Gy may be effective for patients with orbital low grade B-cell lymphomas. Pinnix et al. conducted a retrospective review of 22 patients treated with ultra-low-dose RT for low-grade OAL [20]. They recorded overall response rates of 100% and CR rates of 86%, with no significant acute or late toxicities. Konig et al. analyzed efficacy, toxicity, and relapse rates for indolent orbital lymphoma using ultra-low-dose (n = 7 patients, n = 8 sites) or conventional RT (n = 45 patients, n = 52 sites) [15]. They found response rates (ultra-low-dose vs conventional dose) of 100% versus 98%, acute toxicities of dermatitis/hyperpigmentation (0% vs 79%), and conjunctivitis (0% vs 60%), late toxicities of xerophthalmia (13% vs 40%), and cataract (0% vs 26%), and 2-year local progression-free survival rates of 100% versus 94%. They concluded that ultra-low-dose and conventional radiotherapy were both effective, but ultra-low-dose radiotherapy demonstrated fewer radiation-related side effects than conventional radiotherapy.

But all the above studies were retrospective design. This was the first prospective study investigating treatment efficacy of ultra-low-dose RT in low-grade OAL patients. In this study, 88% of patients had an overall response, 75% achieved a CR, and only 2 patients (12%) had a local relapse after treatment with ultra-low-dose RT. We also documented timings of treatment response and conversions of PR to CR in our study. It should be noted that, all 16 patients responded well to ultra-low-dose RT initially with an average lesion area reduction by 72%. Median time to initial regression was 2.0 months (range 1.0–4.0 months). Response to ultra–low-dose RT was not apparent in one patient at the initial follow-up visit at 2 months, but became apparent and achieved CR at 6 months after RT. One of the two patients who had disease progression lost follow-up after completion of ultra–low-dose RT and developed a relapse 12 months after. The other had a well initial response with lesion area reduced by 63% at 1.5 months after RT. But local recurrence developed with lesion area enlarged to 201.5 mm^2^ at 3 months. From the literatures, little is known about how rapidly a tumor shrinks after radiotherapy. Tsang et al. reported that CR was observed in 30 of 31 patients with ocular adnexal MALT lymphoma at 2–6 months after radiotherapy [21]. Uno et al. reported an initial response rate of 52% with CR at 4–6 weeks after radiotherapy [22]. Our data indicated similar results of quick response to treatment as stated in those published papers. But we emphasized that adequate follow-up time is still neccessary for maximal response or possible recurrence to occur before decisions are made about additional therapy.

Radiographic imaging measurements were used in our study to better evaluate treatment response. We noticed that postoperative lesion area in patients with local recurrence were bigger than that in patients with PR or CR (195.3 ± 13.4 mm^2^ vs. 177.0 ± 78.2 mm^2^ vs. 87.7 ± 31.4 mm^2^, P = 0.003), indicating that patients with smaller lesion might have better response to ultra–low-dose RT. But future study with bigger patient sample would be needed to provide further prognostic value of lesion area to treatment response.

The main limitations of our study were the small patient population, which was in part due to the uncommon nature of OAL, and non-randomized, single-arm features. Future studies of multicenter, randomized clinical trials may lead to stronger evidence to evaluate the efficacy, safety and indications of this potential new treatment.

Compare with conventional dose treatment, ultra–low-dose RT offers distinct benefits of durable local control with shorter treatment duration (2 days as opposed to 12 days), lower treatment expense, and minimal ocular toxic effects. In patients with early-stage OAL and localized lesion, ultra–low-dose RT may offer adequate disease control and maintain long-term good vision function.

In summary, this study confirms that ultra-low-dose RT of 4 Gy in 2 Gy equivalents yields adequate local disease control and long-term survival in low-grade OAL with no significant acute or late toxicities. Long-term observation with careful attention to local relapse is necessary. Given the indolent nature of the disease and the low levels of toxicity associated with lower dose orbital RT, this regimen remains our favoured approach to the management of localized low-grade OAL.

## Supplementary Information

**Additional file 1. Figure S1. **Representative Case 3: A 52-year-old man demonstrated eye redness and ptosis in the right eye showing (a) epibulbar, salmon-like mass superiorly and (b) a dominant enhancing eyelid mass measuring 85.5mm2 on coronal MRI imaging. Biopsy revealed low-grade mature B cell lymphoma of mucosa-associated lymphoid tissue (MALT) type. (c) He was treated with ultra-low-dose radiotherapy using wedges to create a homogeneous dose distribution. (d) Complete response was achieved at 4 months with complete resolution of conjunctival lesion. (e-f) He had a partial response to treatment at 2 months after completion of radiotherapy with lesion area reduced to 52.4mm2 (e) and no detectable lesion on MRI imaging (f). No evidence of recurrence was observed at an additional 14 months of follow-up. **Figure S2.** Representative Case 9: A 52-year-old man was referred for an enlarging lower eyelid mass in the left eye. Biopsy revealed low-grade mature B cell lymphoma of mucosa-associated lymphoid tissue (MALT) type. (a) Axial MRI imaging showed a dominant enhancing eyelid and orbit mass measuring 185.8mm2 after excisional biopsy. (b) He was treated with ultra-low-dose radiotherapy using wedges to create a homogeneous dose distribution. (c) He had a partial response to treatment at 1 months after completion of radiotherapy with lesion area reduced to 68.5mm2. (d) Local recurrence developed with lesion area enlarged to 201.5 mm2 at 3 months after radiotherapy. The patient was successfully retreated with conventional dose radiation (24 Gy in 12 fractions).

## Data Availability

The data sets supporting the results of this article are included within the article and its additional files.
