# Supplementary material for: Ultra-low-dose radiotherapy in the treatment of ocular adnexal lymphoma: a prospective study
Source: Radiat Oncol. 2022 Dec 20;17:208. doi: 10.1186/s13014-022-02180-6 (PMC9764465; doi:10.1186/s13014-022-02180-6)
Supplement: Supplementary file 1 — Additional file 1. Figure S1. Representative Case 3: A 52-year-old man demonstrated eye redness and ptosis in the right eye showing (a) epibulbar, salmon-like mass superiorly and (b) a dominant enhancing eyelid mass measuring 85.5mm2 on coronal MRI imaging. Biopsy revealed low-grade mature B cell lymphoma of mucosa-associated lymphoid tissue (MALT) type. (c) He was treated with ultra-low-dose radiotherapy using wedges to create a homogeneous dose distribution. (d) Complete response was achieved at 4 months with complete resolution of conjunctival lesion. (e-f) He had a partial response to treatment at 2 months after completion of radiotherapy with lesion area reduced to 52.4mm2 (e) and no detectable lesion on MRI imaging (f). No evidence of recurrence was observed at an additional 14 months of follow-up. Figure S2. Representative Case 9: A 52-year-old man was referred for an enlarging lower eyelid mass in the left eye. Biopsy revealed low-grade mature B cell lymphoma of mucosa-associated lymphoid tissue (MALT) type. (a) Axial MRI imaging showed a dominant enhancing eyelid and orbit mass measuring 185.8mm2 after excisional biopsy. (b) He was treated with ultra-low-dose radiotherapy using wedges to create a homogeneous dose distribution. (c) He had a partial response to treatment at 1 months after completion of radiotherapy with lesion area reduced to 68.5mm2. (d) Local recurrence developed with lesion area enlarged to 201.5 mm2 at 3 months after radiotherapy. The patient was successfully retreated with conventional dose radiation (24 Gy in 12 fractions). [file 13014_2022_2180_MOESM1_ESM.docx]

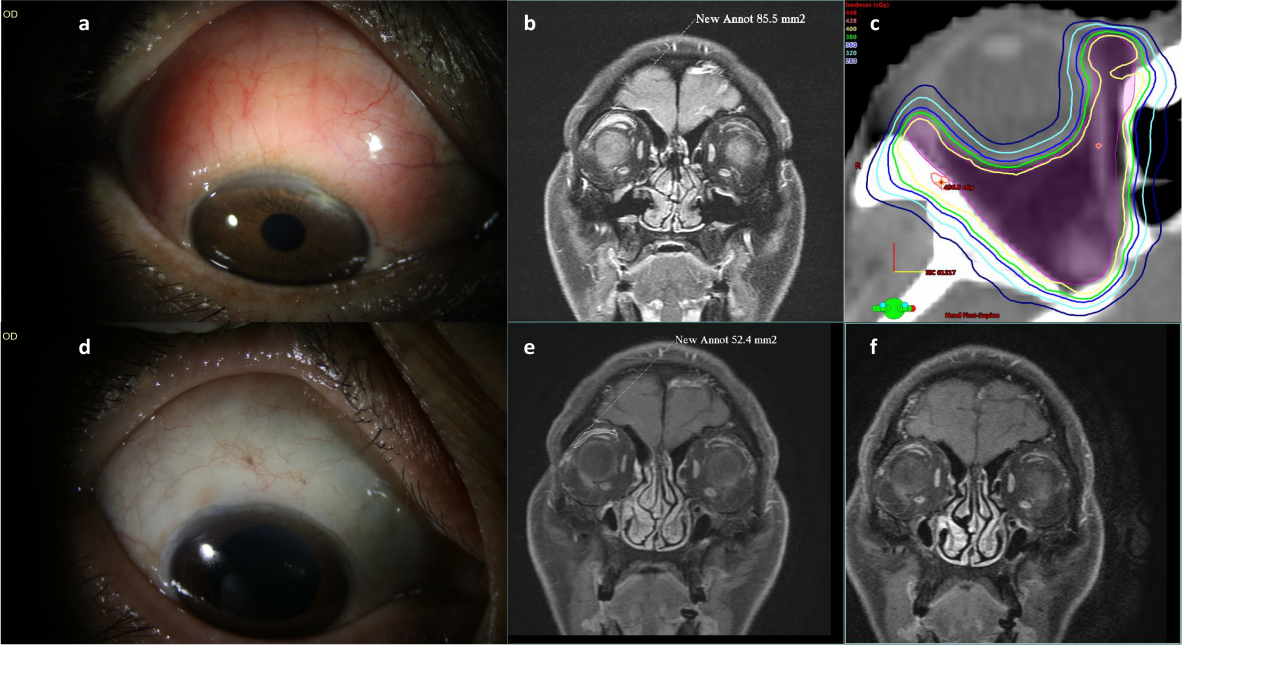


**Figure S1**. Representative Case 3: A 52-year-old man demonstrated eye redness and ptosis in the right eye showing (a) epibulbar, salmon-like mass superiorly and (b) a dominant enhancing eyelid mass measuring 85.5mm2 on coronal MRI imaging. Biopsy revealed low-grade mature B cell lymphoma of mucosa-associated lymphoid tissue (MALT) type. (c) He was treated with ultra-low-dose radiotherapy using wedges to create a homogeneous dose distribution. (d) Complete response was achieved at 4 months with complete resolution of conjunctival lesion. (e-f) He had a partial response to treatment at 2 months after completion of radiotherapy with lesion area reduced to 52.4mm2 (e) and no detectable lesion on MRI imaging (f). No evidence of recurrence was observed at an additional 14 months of follow-up.

**Figure S2**. Representative Case 9: A 52-year-old man was referred for an enlarging lower eyelid mass in the left eye. Biopsy revealed low-grade mature B cell lymphoma of mucosa-associated lymphoid tissue (MALT) type. (a) Axial MRI imaging showed a dominant enhancing eyelid and orbit mass measuring 185.8mm2 after excisional biopsy. (b) He was treated with ultra-low-dose radiotherapy using wedges to create a homogeneous dose distribution. (c) He had a partial response to treatment at 1 months after completion of radiotherapy with lesion area reduced to 68.5mm2. (d) Local recurrence developed with lesion area enlarged to 201.5 mm2 at 3 months after radiotherapy. The patient was successfully retreated with conventional dose radiation (24 Gy in 12 fractions).
